# Henry Overton Wills

**Published:** 1911-09

**Authors:** 


### HENRY OVERTON WILLS.

We have to deplore the loss of the first Chancellor of the University, Mr. H. Overton Wills, who died, deeply regretted by his fellow-citizens, on September 4th. His generosity was nowhere more appreciated than by the University of Bristol, not only on account of the liberality of his donations, but also by reason of their timeliness; for it is not wide of the mark to say that his gift of £100,000 came at a time when hopes were beginning to droop.

Mr. Wills's Chancellorship was all too brief, but it lasted long enough to establish the correctness of his judgment that Bristol was ready for a University. His memory will live through successive generations as the chief founder, and though hereafter munificence equal to his may replenish the coffers of the University, it will never be forgotten that he brought the University into being.

